# Domain-specific perceptual grouping of human dyads: evidence from autistic trait profiles

**DOI:** 10.1038/s41598-026-61597-1

**Published:** 2026-07-10

**Authors:** Bethany E. Richards, James T. Enns, Kim Shapiro, Liuba Papeo, Ana Pesquita

**Affiliations:** 1 School of Psychology, Centre for Human Brain Health, University of Birmingham, Birmingham, UK; 2 Department of Psychology, University of British Columbia, Vancouver, BC Canada; 3 Institut des Sciences Cognitives—Marc Jeannerod, UMR5229, Centre National de la Recherche Scientifique (CNRS) and Université Claude Bernard, Lyon 1, France; 4 Institute of Health and Neurodevelopment (IHN), Aston University, Birmingham, UK

**Keywords:** Perceptual grouping, Social cognition, Gestalt, Autism, Autistic traits, Social perception, Neuroscience, Psychology, Psychology

## Abstract

A longstanding debate in social cognition centres on distinguishing between cognitive processes that are specialised for social content and those that function across multiple domains. Recent research shows that the visual processing of human figures in apparent social interactions is marked by early perceptual grouping. However, whether perceptual grouping of social scenes occurs through a social-specific or domain-general mechanism remains unclear. We examined this question by investigating the relationship between individual differences in social aptitude and perceptual grouping in geometric and social domains. Participants (*n* = 172) completed selective and integrative attention tasks featuring visual displays of geometric and human dyads. Their accuracy and reaction times were measured, and individual social traits were assessed using Autism Quotient Questionnaire subscales. Both geometric and social stimuli showed significant perceptual grouping effects, characterised by faster processing under integrative attention and slower processing under selective attention. Notably, individuals with higher social autistic traits tended to show selective difficulties in social perceptual grouping while maintaining intact geometric grouping, a pattern consistent with domain-specific processes in social scene processing. These findings suggest that even fundamental visual processes may be specifically tuned for social perception, with potential implications for understanding individual differences in social cognition.

**Supplementary Information:**

The online version contains supplementary material available at https://doi.org/10.1038/s41598-026-61597-1.

## Introduction

Recent evidence suggests that the visual system’s early computational stages play a more fundamental role in processing social interactions than previously thought^1^. A compelling series of studies demonstrate that, when viewing two human figures facing each other, the visual system prioritises processing the unified pair over the individual bodies, reflecting a form of perceptual grouping^2–5^. These findings indicate that basic visual processes might be sufficient to detect social relationships between individuals, independent of higher-order cognitive mechanisms^6^. However, a critical question remains: are the perceptual grouping mechanisms for human dyads domain-general (operating similarly across various configural stimuli, including geometric gestalts) or domain-specific (uniquely adapted in the present case for processing social information)? To address this question, we examine the relationship between individual differences in social aptitude and perceptual grouping in both social and non-social domains.

The perceptual grouping of social stimuli was first established through the ‘two-body inversion effect,’ which demonstrates that - in a masked visual categorisation task – inverted facing body dyads are harder to recognise than inverted non-facing dyads^3,5^. This effect is observed specifically in human-human dyads and does not extend to human-object or object-object configurations^3,5^. Further evidence comes from the ‘body inferiority vs. dyad superiority effect,’ showing that while facing dyads are detected more rapidly than non-facing dyads, identifying individual bodies within facing dyads requires more time^4^. These findings collectively show that human-dyads portray the hallmark of perceptual grouping: enhanced processing of unified social configurations and delayed access to individual elements.

To address the question of whether the perceptual grouping of social dyads relies on domain-general processes—similar to those governing gestalt perception—or social-specific mechanisms, researchers have proposed examining how individual differences in social aptitude relate to social grouping mechanisms^7,8^. Later evidence showed that individuals with higher autistic traits revealed reduced sensitivity to social grouping in facing dyads^2^. However, this observation alone cannot establish domain specificity for two reasons. Firstly, the Autism-Spectrum Quotient (AQ;^9^ encompasses broad individual differences beyond social aptitude, including attention and imagination, making it difficult to isolate whether social ability specifically drives the relationship with visual perceptual processing. Secondly, without comparing social and non-social perceptual grouping, we cannot determine whether this relationship is specific to social processing or reflects a general perceptual tendency^10–14^. The current study addresses these limitations in two ways. Firstly, we focus specifically on the social components of the AQ—social skill and communication—to isolate the role of social abilities. Secondly, we examine whether the relationship between social autistic traits and configurational perception is domain-specific by examining perceptual grouping sensitivity to both social and non-social stimuli.

**Fig. 1:**
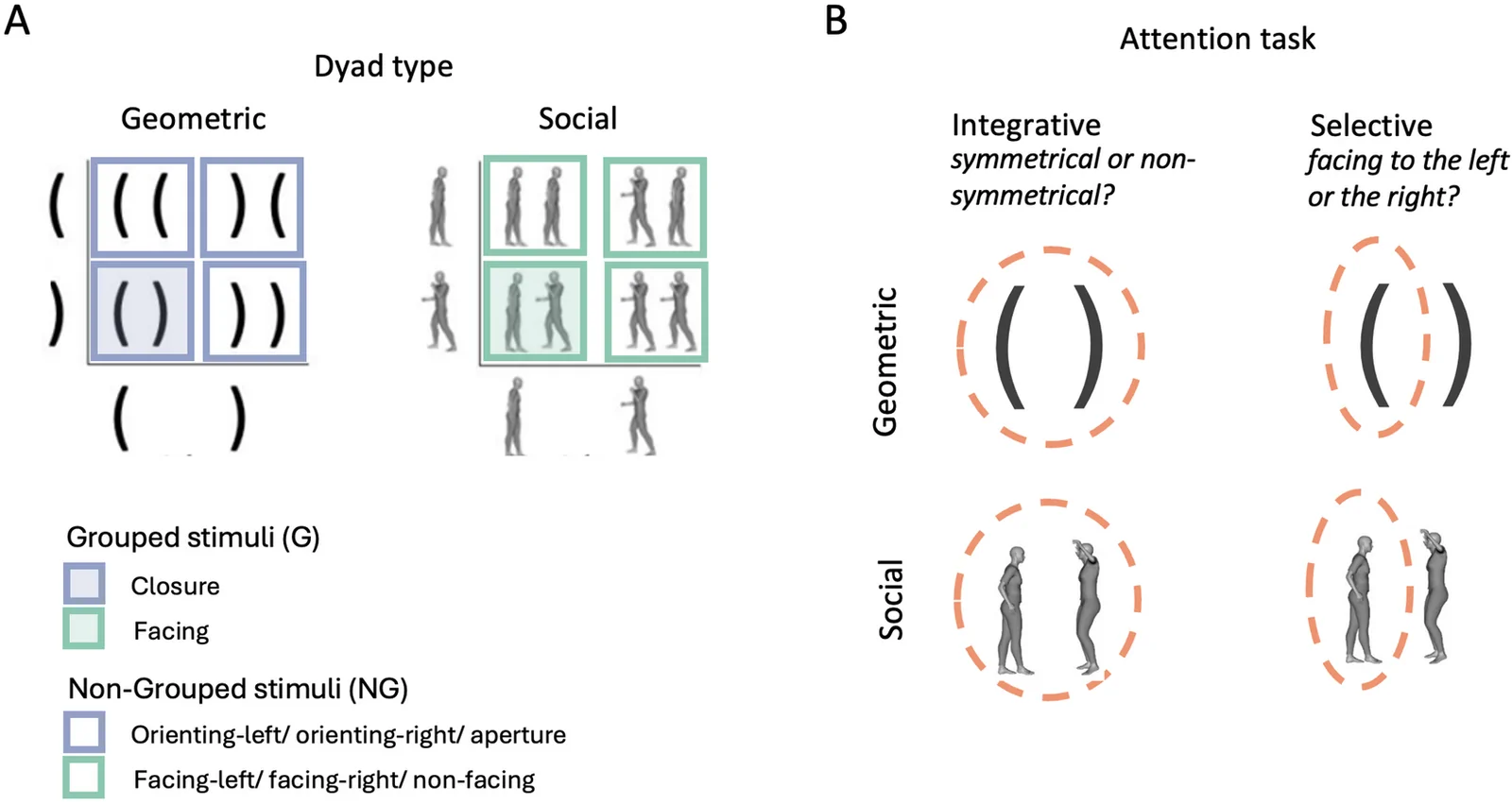
Geometric and social dyad stimuli in selective and integrative attention tasks. (**A**) Stimuli construction diagram. Following Enns and Girgus’^15^ original design, we created geometric configurations using paired parentheses at varying display orientations. When the parentheses are oriented to form closure, they create emergent perceptual characteristics that lead to grouping (geometric grouped stimuli). For the social stimuli, we created analogous configurations using human figures. When the human figures are positioned face-to-face, they suggest social interaction, hypothesised to lead to perceptual grouping (socially grouped stimuli). To create non-grouped stimuli in both domains, we systematically varied the orientation and location of the elements. This ensures a balanced stimulus set with matched grouped and non-grouped configurations across geometric and social domains. (**B**) Participants completed two distinct attention tasks for both stimulus types. In the selective attention task, participants focused on a single element while ignoring others. In the integrative attention task, participants analysed the relationship between elements. The dashed circles (shown for illustration only) highlight the key difference between attention tasks: selective attention requires the processing of an individual element. In contrast, integrative attention requires processing the relationship between elements.

Figure 1A illustrates our adaptation of Enns and Girgus’^15^ methodology to study perceptual grouping. In the original study, geometric displays were created using paired parentheses at varying display locations. When the parentheses were oriented to create a closure (a well-established gestalt principle), the configuration elicited perceptual grouping. In this study, we created an analogous configuration using human figures. When two human-figures are positioned face-to-face, they create a socially meaningful configuration, hypothesised to induce perceptual grouping. We termed closure geometric displays and facing human dyads as ‘grouped’ stimuli and created matched ‘non-grouped’ stimuli by systematically varying the orientation of elements. Participants respond to grouped and non-grouped displays of geometric and social stimuli under two attention tasks. As illustrated in Fig. 1B, in the selective attention task, participants focused on a single element while actively ignoring others (e.g., indicating whether the left figure faced left or right). In the integrative attention task, participants analysed the relationship between elements (e.g., judging whether the configuration was symmetrical or non-symmetrical).

We hypothesised that both geometric and social stimuli would lead to slower responses in the *selective* task but faster responses in the *integrative* task—suggesting prioritised processing of holistic configurations over individual elements^16,17^. Furthermore, we considered two competing hypotheses regarding the relationship between perceptual grouping and social aptitude. The *domain-specific hypothesis* predicts that participants with higher social autistic traits will show reduced perceptual grouping only for social stimuli, with no relationship between social traits and geometric grouping. Conversely, the *domain-general hypothesis* predicts similar associations between social autistic traits and perceptual grouping across social and geometric stimuli.

## Methods

### Participants

182 participants (151 female, 30 male, 1 non-binary) aged 18–27 years (*M*_age_ = 19.27 years, *SD*_age_ = 1.33) were recruited from the University of Birmingham’s student population and invited to complete the experiment online. The distribution of AQ scores in our participant sample showed the same positive skew as Baron-Cohen et al.‘s (2001) general population sample. Our participants’ total AQ score (*M* = 18.3, *SD* = 7.3) closely matched their student population (*M* = 17.6, *SD* = 6.4), as did all subscale scores. Our sample’s scores for Social Skill (*M* = 3.0, *SD* = 2.1) and Communication (*M* = 2.9, *SD* = 2.3) were similar to Baron-Cohen et al.‘s sample (*M* = 2.3, *SD* = 2.2 and *M* = 2.9, *SD* = 2.0, respectively). The same pattern held for Imagination (*M* = 2.6, *SD* = 1.8 vs. *M* = 2.5, *SD* = 1.9), Attention to Detail (*M* = 4.9, *SD* = 2.1 vs. *M* = 5.3, *SD* = 2.2), and Attention Switching (*M* = 5.0, *SD* = 2.0 vs. *M* = 4.5, *SD* = 2.0). In our sample, 5.6% of participants scored at or above the clinical cutoff of 32, slightly higher than in Baron-Cohen et al.‘s^9^ student population (1.3–4.6%). These similarities across total AQ scores, subscale distributions, and clinical threshold rates indicate our sample is representative of the typical student population studied in AQ research. Participants received course credits as compensation for their time. Participants had normal or corrected-to-normal vision. This study was approved by the University of Birmingham Science, Technology, Engineering and Mathematics (STEM) ethics committee (ID: ERN_12-1002P). All research was performed in accordance with the Declaration of Helsinki and relevant institutional guidelines. Informed consent was obtained from all participants prior to their inclusion in the study.

### Stimuli

Figure 1 shows the social and geometric stimuli arranged in distinct configurations – facing/closure, non-facing/aperture, orienting-left and orienting-right. A grey-scale 3D human figure was used to compose the social stimuli^2–5,20^. Using GIMP (The GIMP Team, 2020), we duplicated and horizontally flipped each figure by 180° to create the four distinct dyad configurations. We implemented several control measures to minimise the influence of low-level features on integrative versus selective processing. Firstly, we ensured that each figure contained an equal number of pixels, resulting in uniform surface area coverage across all dyad configurations. Secondly, we maintained a consistent distance between figures within each dyad across all configurations. Specifically, we kept 2 degrees of visual angle between the closest external boundaries of each figure. This approach ensured that the distance between the nearest points of the two figures remained comparable across stimuli configurations.

### Experimental design

The experiment followed a 2 (attention task: integrative, selective) x 4 (dyad configuration: facing/closure, non-facing/aperture, orienting right, orienting left) × 2 (dyad type: social, geometric) x 2 (stimuli order: social first, geometric first) repeated-measures design. Participants completed 160 test trials, divided into four experimental blocks (40 trials per block). Participants completed two blocks of each task type, each represented by one block of body dyads and one block of geometric dyads (40 integrative body, 40 selective body, 40 integrative geometric, and 40 selective geometric). Within each block, each dyad configuration was represented by ten trials. The order in which participants completed the integrative and selective attention tasks was counterbalanced. The order in which participants completed body or geometric blocks first was also counterbalanced to minimise order effects.

### Procedure

Participants completed the online experiment remotely via Gorilla™ (www.gorilla.sc), a digital experiment platform. Participants were instructed to sit in a quiet place with no distractions to ensure maximum focus. To ensure standardisation of stimuli size, participants set up their workspace according to two instructions: firstly, participants adjusted their seating distance to be positioned at one arm’s length from their computer screen. Secondly, participants completed a screen calibration procedure in which they were asked to place a credit card on the screen and adjust the image size of a virtual credit card to match the size of their card^21^. This procedure automatically adjusted the stimuli size for each participant’s home setup. After completing the screen calibration, participants began the experiment.

Participants completed both integrative and selective attention tasks. In the integrative attention task, participants were instructed to attend to both figures in the dyad and to indicate if they were symmetrical or non-symmetrical. In the selective attention task, participants were asked to attend to the left figure in the dyad and to indicate if it was facing to the left or the right. At the start of each trial, participants fixated on a central black cross for 1000-1500ms. Following this, the dyad was displayed until participants made their response or until trial timeout at 3000ms. Participants were asked to respond as fast and accurately as possible. Participants were instructed to submit their responses using their index fingers via the ‘Q’ or ‘P’ keys and received visual accuracy feedback after each trial. ‘Q’ and ‘P’ responses were counterbalanced across participants. Participants completed ten practice trials at the start of each task. The attention tasks took approximately 20 minutes to complete.

Before exiting the experiment, participants completed the AQ questionnaire. The AQ is a 50-item self-report scale used to quantify autistic traits^9^. Participants are assessed across five domains: social skill, attention switching, attention to detail, communication, and imagination. Participants rate the extent to which they agree with statements (e.g., “I prefer to do things with others rather than on my own”), using a Likert scale (“Definitely Agree”, “Slightly Agree”, “Slightly Disagree”, and “Definitely Disagree”). Based on their responses, participants received a score from 0 to 10 on each of the five domains measured by this questionnaire, with higher scores indicating higher autistic traits in each domain. An overall score of 32 is recommended as the cut-off for clinical follow-up^9^. In addition to the 50 items, two attention probe questions were included (e.g., “I have taken this questionnaire seriously and answered honestly”) to ensure that participants answered meaningfully. Participants who answered these questions incorrectly or did not complete the AQ were excluded. The AQ scale took approximately 5 min to complete.

### Data preprocessing

Participants were excluded if they met any of the following criteria: incomplete datasets, failed attention check questions (*n* = 5). 5 participants with error rates exceeding three standard deviations above the mean (27.81%) were also excluded. Including the 5 participants excluded due to error rates does not change the pattern of results. The final sample consisted of 172 participants. 6.94% of trials were excluded, comprising incorrect responses (5%) and trials with reaction times below 150ms or beyond three standard deviations from the mean (1.94%). All stimulus materials, data and analysis scripts are publicly available (https://osf.io/32xcr/?view_only=8ef24c1502de4a4cb438c45703134c80).

## Results

To quantify perceptual grouping—characterised by faster responses under integrative attention and slower responses under selective attention^16,17^—we computed perceptual grouping scores for each participant in both geometric and social conditions using Eq. (1).

1 $$\:Perceptual\:grouping\:=\left(\frac{{I}_{NG}-\:{I}_{G}\:}{{I}_{NG}\:+\:{I}_{G}\:}\right)+\left(\frac{{S}_{G}-\:{S}_{NG}\:}{{S}_{G}\:+\:{S}_{NG}\:}\right)$$

In Eq. (1), $\:{I}_{NG}$ corresponds to reaction times for non-grouped stimuli (i.e., aperture/non-facing, orienting-left/facing-left, orienting-right/facing right) in the integrative attention task; $\:{I}_{G}\:$represents reaction times for grouped stimuli in the integrative attention task (i.e., facing/closure); and $\:{S}_{NG}$ and $\:{S}_{G}$ correspond to reaction times for non-grouped and grouped stimuli in the selective attention task respectively. The first term indexes the facilitation of responses to grouped stimuli in the integrative attention task, while the second term quantifies the interference of grouped stimuli in the selective attention task. Their sum yields the perceptual grouping score. This scoring method offers two key advantages over direct reaction time analysis: (1) It integrates facilitation and interference effects into a single, standardised metric of perceptual grouping; (2) It allows straightforward testing of perceptual grouping by examining whether grouping scores reliably exceed zero.

**Fig. 2:**
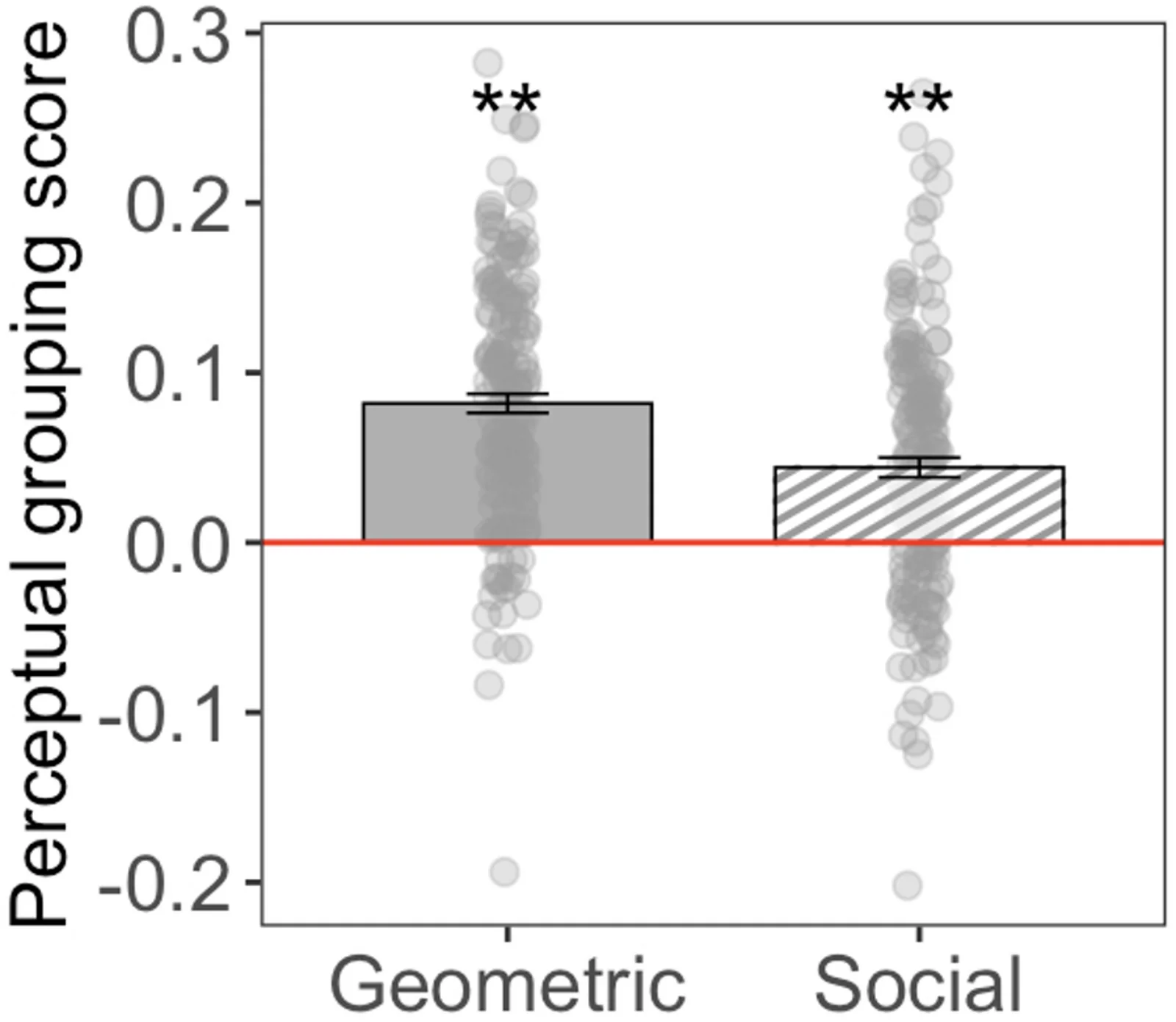
Perceptual grouping of geometric and social and social dyads. Mean perceptual grouping scores for geometric and social dyad stimuli. Reliably positive scores indicate a perceptual grouping effect. Both geometric configurations and social dyads showed significant perceptual grouping effects. Error bars represent standard error of the mean. Data points represent individual participants mean scores. Asterisks indicate significant perceptual grouping effects relative to zero (**p < .001).

Figure 2 shows the perceptual grouping scores for geometric and social figures. We computed Bonferroni-corrected one-sample one-tailed t-tests to determine if perceptual grouping scores were significantly above zero in geometric and social stimuli types. The results indicated significant differences for both geometric (*M* = 0.082, *SD* = 0.074, *t*(171) = 14.57, *p* < .001, *d* = 1.11, 95% confidence interval [CI] = [0.95, Inf]), and social stimuli (*M* = 0.044, *SD* = 0.076, *t*(171) = 7.58, *p* < .001, *d* = 0.58, 95% CI = [0.44, Inf]). Employing a more stringent significance criterion (α = 0.025) did not alter these findings, confirming the robustness of the results. We interpret these findings to demonstrate that both geometric closure and facing social dyads lead to perceptual grouping. A paired-sample t-test (two-tailed) revealed that perceptual grouping effects were significantly stronger for geometric stimuli compared to social dyad stimuli, *t*(171) = 5.44, *p* < .001, *d* = 0.50, 95% CI = [0.29, 0.72]. Direct analysis of the reaction time responses supports the perceptual grouping effects reported above. A pronounced floor effect was present in the error-rate data (*M*=0.05, *SD*=0.04, *range*= [0, 0.22]). In most cells of our experimental design, over 50% of participants made no errors, precluding an interpretable computation of a grouping score based on error-rate measurements. For additional information, see more detailed analysis of RT and error rate in the supplementary material (SM1 and SM2 respectively).

To identify distinct subgroups based on social autistic traits, we applied cross-validated k-means clustering to participants’ AQ scores in social skill and communication dimensions. We used these two subscales directly, rather than a derived social factor, because the factor structure of the AQ remains unclear^18,19^. The two subscales were standardised (z-scored) before clustering to give them equal weight, and k-means was run with 25 random starts and a fixed seed for reproducibility. The number of clusters was selected using the elbow method on the within-cluster sum of squares (WSS) alongside the average silhouette width. The largest reductions in WSS occurred over the first three clusters, accounting for 82.3% of the total reduction and 68.83% of the variance. We assessed the stability of the three-cluster solution using 5-fold cross-validation repeated 10 times, which showed consistent performance across folds (mean stability = 0.51, SD = 0.02; mean silhouette = 0.42, SD = 0.02; mean variance explained = 0.69, SD = 0.01). Full details of cluster selection and validation are reported in Supplementary Material SM3.

**Fig. 3:**
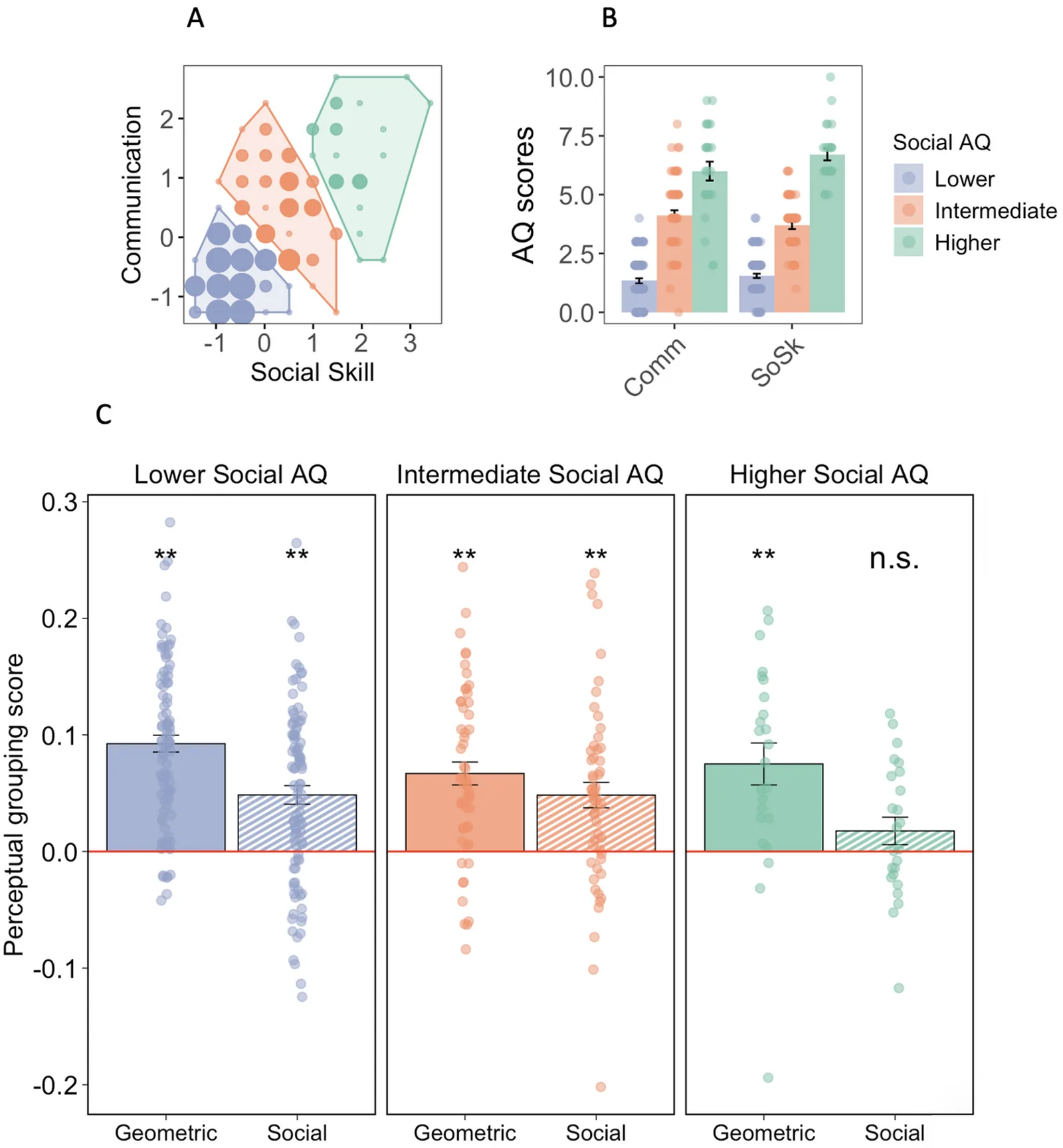
Differential perceptual grouping of geometric and social stimuli reveals distinct processing patterns across social autistic trait subgroups. (**A**) Data-Driven Classification of Social Autistic Traits Reveals Three Distinct Subgroups. Cross-validated k-means clustering analysis of Autism-Spectrum Quotient (AQ) scores in social skill and communication dimensions. Three stable clusters emerged: a higher-scoring subgroup with pronounced social autistic traits (in green), an intermediate with moderate traits (in orange), and a lower-scoring subgroup with minimal traits (in blue). Data points represent individual participants plotted by their social skill and communication scaled scores. The size of the data points indicates the frequency of participants in the same coordinates. (**B**) Mean AQ scores in the Communication and Social Skill dimensions. (**C**) Mean perceptual grouping scores for geometric and social stimuli across three social AQ subgroups (higher, intermediate, and lower). Reliably positive scores indicate the presence of perceptual grouping effects. Error bars represent standard error of the mean. Data points represent individual participant’s scores. Asterisks indicate significant perceptual grouping effects relative to zero (**p < .001)

The data-driven approach described above revealed natural groupings in social traits, avoiding arbitrary cutoff points that might not reflect the true distribution of autistic characteristics in the population. As shown in Fig. 3A,B, a three-cluster solution emerged, revealing a hierarchical pattern of social autistic traits: A lower-scoring social AQ subgroup (*n* = 93) exhibited minimal social skill (*M* = 1.55, *SD* = 0.891) and communication traits (*M* = 1.33, *SD* = 1.05); an intermediate social AQ subgroup (*n* = 55) displayed moderate scores in both dimensions (social skill: *M* = 3.69, *SD* = 1.14; communication: *M* = 4.11, *SD* = 1.67); and a higher-scoring social AQ subgroup (*n* = 24) showed the most pronounced traits in both social skill (*M* = 6.71, *SD* = 1.23) and communication (*M* = 6.00, *SD* = 1.98). See Supplementary Material (SM3) for details on selecting the optimal number of clusters. Whereas the *lower*, *intermediate* and *higher* social aptitude clusters have uneven sizes, with the low-scoring group being the largest and the high-scoring group being notably smaller, this distribution aligns with the expected prevalence of social autistic traits in the general population^9^. We adopted this subgroup design because our hypothesis is group-level: framed within a quasi-neuropsychological logic, it concerns whether individuals whose trait profiles place them at the high end of the social-trait distribution show a selective difference in perceptual grouping, rather than whether grouping varies as a graded function of social traits across the sample. A complementary continuous analysis is reported in the Supplementary Material SM4; it found no significant association, neither linear or quadratic, between social traits and grouping in either domain.

Figure 3C shows the perceptual grouping scores for geometric and social figures in each social AQ subgroup. A mixed ANOVA with stimulus type (geometric, social) as a within-subjects factor and social AQ subgroup (lower, intermediate, higher) as a between-subjects factor, while controlling for order, gender, and age, revealed a significant interaction between stimulus type and social AQ subgroup, *F*(2, 158) = 3.19, *p* = .04, *partial η²* = 0.039. No other main effects or interactions reached statistical significance (all *ps* > 0.18). To follow up on the significant interaction effect, we conducted separate pairwise comparisons for each stimulus type. For geometric stimuli, there were no significant differences in perceptual grouping between the three social AQ subgroups (all *ps* > 0.11). However, for social stimuli, the higher social AQ subgroup showed significantly weaker perceptual grouping compared to the intermediate social AQ subgroup, *t*(158) = -2.15, *p* = .03, d = -0.53, *95% CI* [-1.01, -0.04], and marginally weaker grouping compared to the lower social AQ subgroup, *t*(158) = -1.80, *p* = .074, *d* = -0.41, *95% CI* [-0.86, 0.04]. No significant difference was found between the intermediate and lower subgroups (*p* = .54).

To determine whether perceptual grouping mechanisms were active in each stimulus type and social AQ group, we conducted Bonferroni-corrected one-sample t-tests (one-tailed). In this analysis, scores significantly above zero indicate the presence of perceptual grouping, characterised by faster processing under integrative attention and slower processing under selective attention. The lower social AQ group showed significant perceptual grouping effects for both geometric (*M* = 0.09, *SD* = 0.07, *t*(92) = 12.9, *p* < .001, *d* = 1.33, 95% CI = [0.08, Inf]), and social stimuli (*M* = 0.05, *SD* = 0.08, *t*(92) = 6.05, *p* < .001, *d* = 0.63, 95% CI = [0.04, Inf]). Similarly, in the intermediate social AQ group, significant perceptual grouping effects were observed for both geometric (*M* = 0.07, *SD* = 0.07, *t*(54) = 6.84, *p* < .001, *d* = 0.92, 95% CI = [0.05, Inf]), and social stimuli (*M* = 0.05, *SD* = 0.08, *t*(54) = 4.43, *p* < .001, *d* = 0.6, 95% CI = [0.03, Inf]). For the higher social AQ group, geometric stimuli showed a significant perceptual grouping effect (*M* = 0.08, *SD* = 0.09, *t*(23) = 4.18, *p* = .002, *d* = 0.85, 95% CI = [0.04, Inf]). However, social stimuli did not show a reliable effect (*M* = 0.02, *SD* = 0.06, *t*(23) = 1.51, *p* = .87, 95% CI = [-0.002, Inf]), which suggests a selective difficulty in social perceptual grouping. Employing a more stringent significance criterion (α = 0.025) did not alter this pattern of results.

To examine the apparent absence of social perceptual grouping in the higher social AQ group, we employed the Two One-Sided Tests (TOST) procedure to assess statistical equivalence to zero. Unlike traditional null hypothesis testing, which can only fail to reject differences, TOST allows us to actively test for equivalence by examining whether an effect falls within a predetermined range that would be considered practically equivalent to zero. We set equivalence boundaries corresponding to a medium effect size (*d* = ± 0.5), computed as ± *d* * *SD*_*pooled*_ (equivalence boundaries = ± 0.039). These equivalence boundaries represent the largest effect size that we would consider meaningfully equivalent to zero, with values falling within this range suggesting no practically significant effect. The TOST procedure simultaneously tests two null hypotheses: one that the effect is greater than or equal to the lower boundary, and another that it is less than or equal to the upper bound. Both the lower boundary test (*t*(23) = 4.795, *p* < .001, *d* = 0.98) and upper boundary test (*t*(23) = -1.781, *p* = .04, *d* = -0.36) were significant. The observed social grouping mean (*M* = 0.02) fell within the 95% CI [-0.002, 0.038], indicating that the perceptual grouping effect was statistically equivalent to zero within the specified boundaries. Combined with our previous analyses, this statistical equivalence to zero is consistent with the possibility that individuals with higher social AQ scores experience greater difficulty with social grouping.

## Discussion

Research on perceptual mechanisms and individual differences in social cognition has largely developed along separate paths. Our study bridges these paths by demonstrating that perceptual grouping - a fundamental visual process - operates differently for social versus non-social stimuli depending on individual social traits. Using a quasi-neuropsychological approach, we observed that individuals with higher social autistic traits showed a selective difficulty in grouping social configurations while maintaining intact geometric perceptual grouping, whereas those with lower and intermediate traits demonstrated robust grouping across both domains. This pattern of results supports the existence of domain-specific processes in social scene processing.

The observed dissociation between geometric and social grouping abilities refines current theoretical frameworks, particularly the weak central coherence theory associated with autism spectrum disorder (ASD; hereafter ‘autism’;^22^ and high overall autistic traits as determined by the AQ^10,12–14^. While previous research has documented that autistic people demonstrate enhanced local processing but reduced information integration into coherent wholes, our focused analysis of social skill and communication trait dimensions reveals a more nuanced relationship. The preservation of geometric grouping alongside challenges with social grouping in students with high social autistic traits suggests that general perceptual integration mechanisms can remain intact, with difficulties emerging selectively for social configurations. This domain-specificity is further supported by the intermediate social AQ group’s preserved performance across both domains, indicating a threshold effect where challenges in processing social configurations emerge only at higher levels of social autistic traits.

Two methodological innovations strengthened our investigation: First, we developed a comprehensive perceptual grouping score that measures performance against a theoretical baseline of zero effect, providing a more sensitive way to detect genuine perceptual grouping than traditional reaction time analyses. Second, our data-driven clustering approach to analysing social autistic traits improves upon arbitrary cutoff scores, allowing natural groupings to emerge from the data. The moderate silhouette score and stable cross-validation results support the reliability of these groupings, suggesting potential value for future studies investigating individual differences in social perception. This subgroup approach may also be informative where an effect is concentrated within a particular part of the trait distribution rather than varying smoothly across it, a pattern that a continuous analysis spanning the whole sample can dilute and potentially overlook.

Our findings can be interpreted through Lockwood et al.‘s^23^ influential framework, which proposes that social processing can be specialised at computational, algorithmic, and implementational levels. While the computational principles of perceptual grouping appear shared across domains - evidenced by faster integrative and slower selective attention effects for both stimulus types - the systematic variation in social perceptual grouping with social autistic traits suggests distinct algorithms or specialised neural implementations for social perception. Future algorithmic-level research should quantify capacity limits and temporal dynamics of social versus geometric grouping through visual search paradigms^24,25^. At the implementation level, our findings sit alongside neuroimaging work. Facing dyads appear to engage body-selective occipitotemporal cortex, where they may be represented as enhanced configurations^2,26^, and this processing has been linked to the left extrastriate body area, whose disruption by transcranial magnetic stimulation has been reported to reduce the two-body inversion effect^27^. Whether our social-geometric dissociation maps onto distinct neural circuits is a question for future work. Related findings beyond facing bodies, and from other paradigms, are consistent with this account. Joint-attention dyads may likewise be bound into a single perceptual unit, indexed by a reduced inversion effect that was weaker for non-social directional stimuli^28^, and in a continuous performance task autistic adults showed reduced P300 and contingent negative variation amplitudes for faces but not letters^29^. A social-specific dissociation of this kind, recurring across tasks, measures, and populations, may lend some support to the idea of specialised social processing.

Our research on perceptual grouping domain-specificity and its relationship with social traits should be extended in two directions. The first concerns the robustness of the present finding. The selective social grouping difference we observed in the higher social AQ group rests on a modest subgroup size, which reflects the low prevalence of high social autistic traits in an unselected student sample. Future work should seek to replicate it in larger samples, ideally using targeted recruitment to enrich the upper end of the social-trait distribution, where the effect is concentrated. The second concerns generalisation beyond elevated traits. Extending this work to diverse clinical populations would deepen our understanding of social perceptual organisation: studies with autistic participants could determine whether the patterns observed in individuals with elevated autistic traits generalise to clinical populations, and examining additional neuropsychological profiles characterised by atypical social processing, such as schizophrenia, Williams syndrome, and social anxiety disorder, would help map the broader relationship between social abilities and perceptual organisation. This approach would clarify whether difficulties in social perceptual grouping represent a common characteristic across conditions affecting social cognition or manifest uniquely across different neuropsychological profiles.

## Supplementary Information

Below is the link to the electronic supplementary material.

Supplementary Material 1

## Data Availability

All stimulus materials, data and analysis scripts are publicly available (https://doi.org/10.17605/OSF.IO/UJG3R).
