# Supplementary material for: Domain-specific perceptual grouping of human dyads: evidence from autistic trait profiles
Source: Sci Rep. 2026 Jul 10;16:31750. doi: 10.1038/s41598-026-61597-1 (PMC13654802; doi:10.1038/s41598-026-61597-1)
Supplement: Supplementary file 1 [file 41598_2026_61597_MOESM1_ESM.pdf]

Supplementary Material 1 (SM1)

Figure SM1-1 summarises reaction time data across all experimental conditions, showing mean values for each stimulus type and configuration in both attention tasks. We conducted a 4-way repeated-measures ANOVA (Greenhouse-Geisser corrected) to examine the effects of task (integrative vs. selective attention), configuration (facing, facing-left, facing-right, non-facing), and stimuli type (human vs. geometric) on response times. The analysis revealed significant main effects for task: $F(1, 171) = 640.31$, $p < .001$; configuration: $F(3, 727.56) = 11.95$, $p < .001$; and stimuli type: $F(1, 337.81) = 71.51$, $p < .001$. Significant two-way interactions were found between task and configuration: $F(3, 726.18) = 77.60$, $p < .001$; and task and stimuli type: $F(1, 338.09) = 96.57$, $p < .001$. The two-way interaction between configuration and stimuli type was not significant: $F(3, 654.48) = 0.65$, $p = .584$. Crucially, we observed a significant three-way interaction between task, configuration, and stimuli type: $F(3, 647.51) = 7.14$, $p < .001$. This indicates that response patterns across configurations varied depending on both task and stimulus type. To further analyse this interaction, we split the data by stimulus type.

Geometric Stimuli

For geometric stimuli, a 2-way repeated-measures ANOVA (Greenhouse-Geisser corrected) revealed significant main effects of task: $F(1, 171) = 293$, $p < .001$, ges = .25 and configuration: $F(3, 513) = 4.84$, $p = .002$, ges = .002. A significant task × configuration interaction was also observed: $F(3, 513) = 69.6$, $p < .001$, ges = .033. Follow-up analyses by task showed significant main effects of configuration in both the integrative attention task, $F(2.82, 482) = 37.9$, $p < .001$, ges = .045, and the selective attention task, $F(2.83, 483) = 34.6$, $p < .001$, ges = .024. Bonferroni-corrected pairwise comparisons for the integrative attention task showed that participants responded significantly faster to facing configurations (M = 566 ms, SD = 95.1) compared to facing-left (M = 618 ms, SD = 110), $t(171) = -8.79$, $p <$

.001; facing-right (*M* = 620 ms, *SD* = 116), $t(171) = -9.34$, $p < .001$; and non-facing configurations (*M* = 624 ms, *SD* = 114), $t(171) = -9.50$, $p < .001$. No significant differences were found among facing-left, facing-right, and non-facing configurations (all $p > .05$). In the selective attention task, participants responded significantly slower to facing configurations (*M* = 511 ms, *SD* = 116) compared to facing-left (*M* = 469 ms, *SD* = 98.4), $t(171) = 8.79$, $p < .001$; facing-right (*M* = 472 ms, *SD* = 107), $t(171) = 7.39$, $p < .001$; and non-facing configurations (*M* = 482 ms, *SD* = 99.9), $t(171) = 6.79$, $p < .001$. Additionally, facing-left configurations elicited significantly faster responses than non-facing configurations, $t(171) = -3.27$, $p = .008$, while the difference between facing-right and non-facing configurations was not significant after correction ($p = .215$).

Social Stimuli

For human dyads, a 2-way repeated-measures ANOVA (Greenhouse-Geisser corrected) revealed significant main effects of task: $F(1, 171) = 653$, $p < .001$, ges = .425 and configuration: $F(3, 513) = 8.36$, $p < .001$, ges = .004, with a significant task × configuration interaction: $F(2.88, 492) = 26.1$, $p < .001$, ges = .013. Follow-up analyses by task showed significant main effects of configuration in both the integrative attention task, $F(2.84, 486) = 16.9$, $p < .001$, ges = .02, and the selective attention task, $F(2.75, 471) = 18.4$, $p < .001$, ges = .013. Bonferroni-corrected pairwise comparisons for the integrative attention task showed that participants responded significantly faster to facing configurations (*M* = 646 ms, *SD* = 124) compared to facing-left (*M* = 693 ms, *SD* = 145), $t(171) = -5.85$, $p < .001$; facing-right (*M* = 689 ms, *SD* = 126), $t(171) = -6.23$, $p < .001$; and non-facing (*M* = 686 ms, *SD* = 134), $t(171) = -5.57$, $p < .001$. No significant differences were found between facing-left, facing-right, and non-facing configurations (all $p > .05$). In the selective attention task, participants responded significantly slower to facing configurations (*M* = 488 ms, *SD* = 105) compared to facing-left (*M* = 463 ms, *SD* = 92.4), $t(171) = 5.91$, $p < .001$; and facing-right (*M* = 471 ms, *SD* = 98.3), $t(171) = 3.87$, $p < .001$; but not compared to non-facing configurations (*M* = 491 ms, *SD* = 98.6), $p = .513$. The non-facing configuration elicited

significantly slower responses than facing-left, $t(171) = -6.86$, $p < .001$; and facing-right, $t(171) = -4.09$, $p < .001$, configurations.

These results across both stimulus types reveal a consistent pattern of perceptual grouping for facing configurations. In the integrative attention task, responses to facing configurations were faster than to all other configurations, while in the selective attention task, responses to facing configurations were generally slower. This consistency suggests that the perceptual grouping effect occurs with both geometric and social stimuli.

Figure SM1-1

Mean reaction time data across all experimental conditions

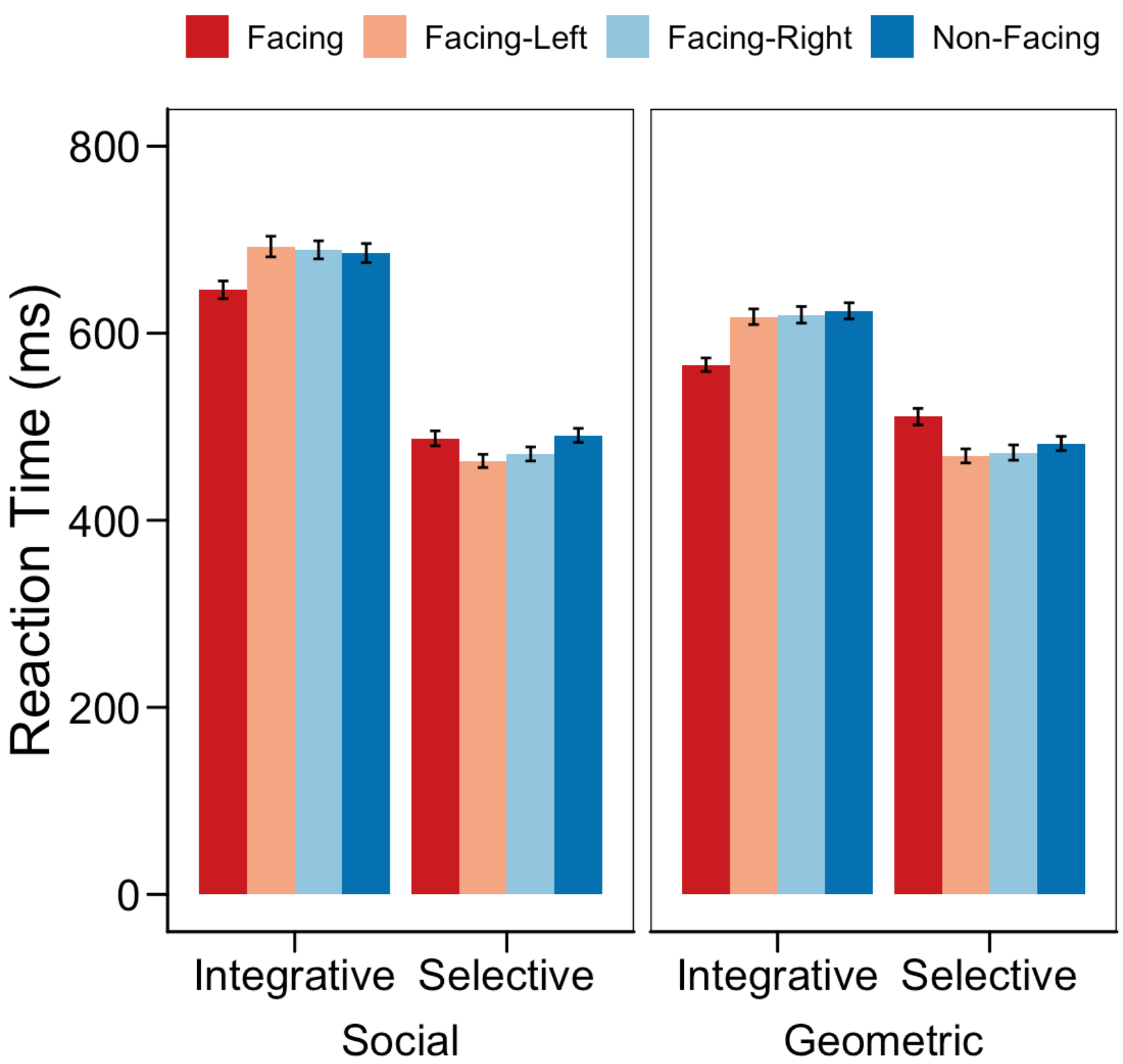

Note. Mean reaction times (in milliseconds) for geometric and social stimuli across four configurations (facing, facing-left, facing-right, and non-facing) in both selective and integrative attention tasks. Error bars represent the standard error of the mean. Faster responses to facing configurations in the integrative task and slower responses in the selective task provide evidence for perceptual grouping effects with both stimulus types. Data points represent individual participants plotted by their social skill and communication scaled scores.

Supplementary Material 2 (SM2)

Figure SM2-1 summarises error rates across all experimental conditions, showing mean values for each stimulus type and configuration in both attention tasks. We conducted a 4-way repeated-measures ANOVA (Greenhouse-Geisser corrected) to examine the effects of task (integrative vs. selective attention), configuration (facing, facing-left, facing-right, non-facing), and stimuli type (human vs. geometric) on error rates. We found significant main effects of task ($F(1, 171.00) = 68.30$, $p < 0.001$) and configuration ($F(3, 512.97) = 22.02$, $p < 0.001$), but not dyad stimuli ($F(1, 342.00) = 0.15$, $p = 0.699$). The interaction between task and configuration was significant ($F(3, 1538.97) = 18.62$, $p < 0.001$), as was the interaction between task and dyad stimuli ($F(1, 342.00) = 5.82$, $p = 0.016$). We also found a significant three-way interaction between task, configuration, and dyad stimuli ($F(3, 1538.97) = 2.71$, $p = 0.044$). This indicates that error rate patterns across configurations varied depending on both task and stimulus type. To further analyse this interaction, we split the data by stimulus type.

Geometric Stimuli

For geometric stimuli, a 2-way repeated-measures ANOVA (Greenhouse-Geisser corrected) revealed significant main effects of task ($F(1, 171) = 28.6$, $p < 0.001$, $\eta^2 = 0.028$) and configuration ($F(2.72, 465) = 15.4$, $p < 0.001$, $\eta^2 = 0.024$). Furthermore, we found a significant interaction between task and configuration ($F(2.88, 493) = 16.6$, $p < 0.001$, $\eta^2 = 0.027$), indicating that the effect of configuration on error rates differed between integrative and selective attention tasks when participants responded to geometric stimuli. Follow-up analyses by task showed significant main effects of configuration in both the integrative attention task, $F(2.6, 444) = 20.2$, $p < .001$, ges = .067, and the selective attention task, $F(2.62, 448) = 8.77$, $p < .001$, ges = .025. Bonferroni-corrected pairwise comparisons for the integrative attention task showed that participants responded with significantly lower error rates in the facing ($M = 0.044$, $SD = 0.076$), facing-left ($M = 0.052$, $SD = 0.100$), and facing-right ($M = 0.049$, $SD = 0.080$) configurations compared to the non-facing condition ($M =$

0.106, *SD* = 0.120; all $p < .0001$) for geometric stimuli. In the selective attention task with geometric stimuli, facing configurations (*M* = 0.054, *SD* = 0.108) showed significantly higher error rates compared to facing-left (*M* = 0.019, *SD* = 0.043; $p < .001$) and facing-right (*M* = 0.026, *SD* = 0.078; $p < .01$) configurations, while differences between other configurations were not significant (non-facing: *M* = 0.034, *SD* = 0.078; all $p > .05$).

Social Stimuli

For social stimuli, a 2-way repeated-measures ANOVA (Greenhouse-Geisser corrected) revealed significant main effects of task ($F(1, 171) = 53.8$, $p < 0.001$, $\eta^2 = 0.055$) and configuration ($F(3, 513) = 8.96$, $p < 0.001$, $\eta^2 = 0.012$). Furthermore, we found a significant interaction between task and configuration ($F(2.84, 486) = 4.65$, $p < 0.01$, $\eta^2 = 0.007$), indicating that the effect of configuration on error rates differed between integrative and selective attention tasks when participants responded to social stimuli. Follow-up analyses by task showed significant main effects of configuration in both the integrative attention task, $F(2.86, 489) = 6.56$, $p < .001$, ges = .018, and the selective attention task, $F(2.5, 427) = 7.51$, $p < .001$, ges = .024. Bonferroni-corrected pairwise comparisons for the integrative attention task showed that participants responded with significantly lower error rates in the facing (*M* = 0.055, *SD* = 0.117; $p < .0001$) and facing-left configurations (*M* = 0.066, *SD* = 0.122; $p < .05$) compared to the non-facing condition (*M* = 0.100, *SD* = 0.132), with no significant difference between facing-right (*M* = 0.072, *SD* = 0.129; $p = .073$) and non-facing configurations. In the selective attention task with social stimuli, facing configurations (*M* = 0.034, *SD* = 0.073) showed significantly higher error rates compared to facing-right configurations (*M* = 0.015, *SD* = 0.045; $p < .01$), while facing-left (*M* = 0.016, *SD* = 0.060) and facing-right configurations showed significantly lower error rates compared to non-facing configurations (*M* = 0.037, *SD* = 0.069; both $p < .001$).

Figure SM2-1

Error rates across all experimental conditions

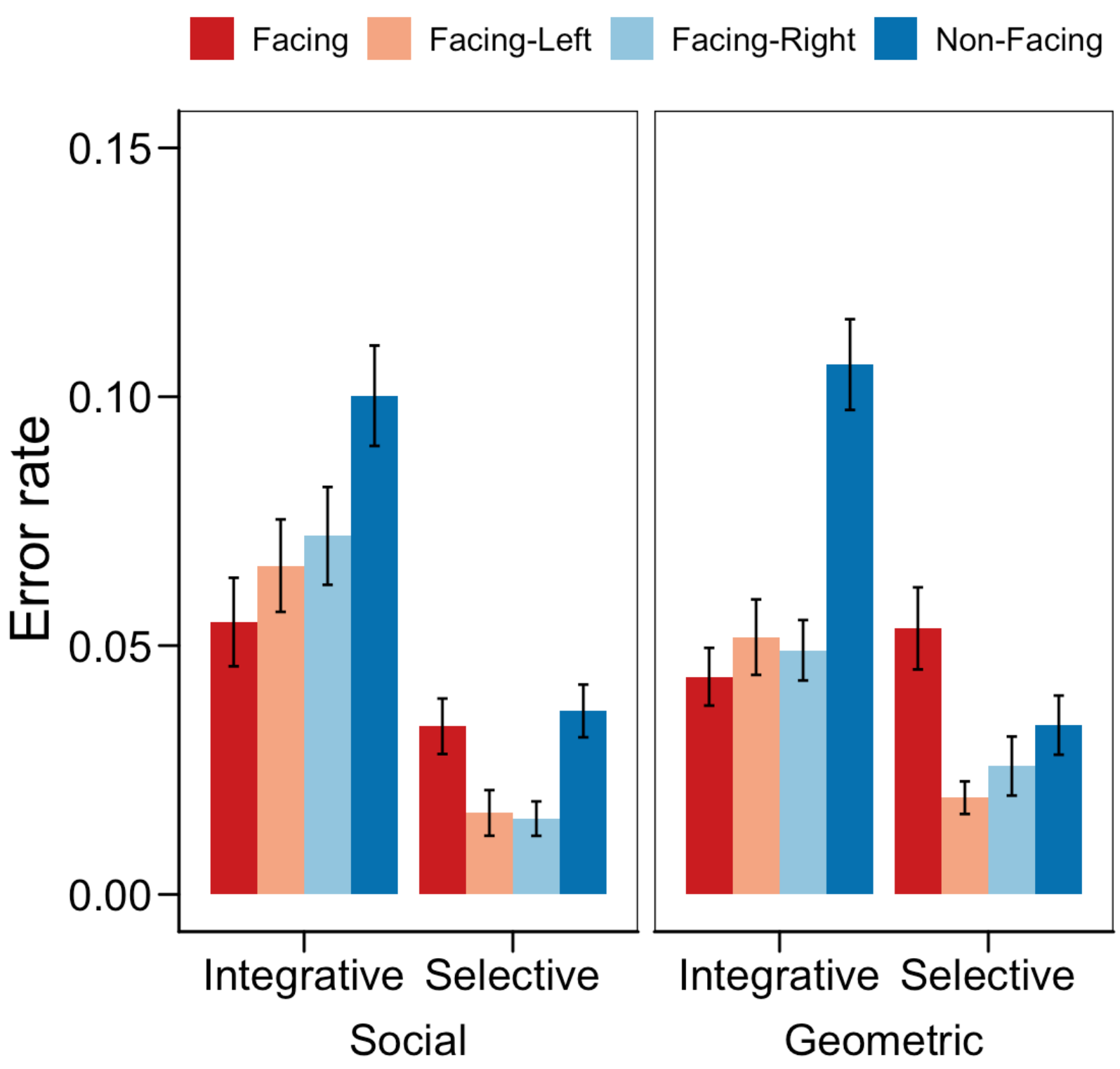

Note. Error rates for geometric and social stimuli across four configurations (facing, facing-left, facing-right, and non-facing) in both selective and integrative attention tasks. Error bars represent the standard error of the mean. Data points represent individual participants plotted by their social skill and communication scaled scores.

Supplementary Material 3 (SM3)

Autism Quotient (AQ) Assessment The AQ is a 50-item self-report scale used to quantify autistic traits (Baron-Cohen et al., 2001). Participants are assessed across five domains: social skill, attention switching, attention to detail, communication, and imagination. Participants rate the extent to which they agree with statements (e.g., "I prefer to do things with others rather than on my own"), using a Likert scale ("Definitely Agree", "Slightly Agree", "Slightly Disagree", and "Definitely Disagree"). Based on their responses, participants received a score from 0-10 on each of the five domains measured by this questionnaire, with higher scores indicating higher autistic traits in each domain. An overall score of 32 is recommended as the cut-off for clinical follow-up (Baron-Cohen et al., 2001). In addition to the 50 items, two attention probe questions were included (e.g., "I have taken this questionnaire seriously and answered honestly") to ensure that participants answered meaningfully. Participants who answered these questions incorrectly or did not complete the AQ were excluded. The AQ scale took approximately 5 minutes to complete.

Sample Characteristics and Representativeness The distribution of AQ scores in our participant sample showed the same positive skew as Baron-Cohen et al.'s (2001) general population sample. Our participants' total AQ score (M = 18.3, SD = 7.3) closely matched their student population (M = 17.6, SD = 6.4), as did all subscale scores. Our sample's scores for Social Skill (M = 3.0, SD = 2.1) and Communication (M = 2.9, SD = 2.3) were similar to Baron-Cohen et al.'s sample (M = 2.3, SD = 2.2 and M = 2.9, SD = 2.0, respectively). The same pattern held for Imagination (M = 2.6, SD = 1.8 vs. M = 2.5, SD = 1.9), Attention to Detail (M = 4.9, SD = 2.1 vs. M = 5.3, SD = 2.2), and Attention Switching (M = 5.0, SD = 2.0 vs. M = 4.5, SD = 2.0). In our sample, 5.6% of participants scored at or above the clinical cutoff of 32, slightly higher than in Baron-Cohen et al.'s student population (1.3-4.6%). These similarities across total AQ scores, subscale distributions, and clinical threshold rates indicate our sample is representative of the typical student population studied

in AQ research. Our analysis focuses specifically on the social components of the AQ—social skill and communication—as these subscales most directly assess social aptitude and are most theoretically relevant to our investigation of domain-specific perceptual grouping in social contexts.

Cluster Analysis and Validation The optimal number of social aptitude clusters (k = 3) was determined through multiple quantitative criteria. The elbow method revealed substantial reductions in the within-cluster sum of squares (WSS), with the most dramatic decreases occurring in the first three clusters (Figure SM3-1). Specifically, WSS decreased by 57.8% from k = 1 to k = 2 and 24.5% from k = 2 to k = 3, capturing 82.3% of the total WSS reduction (Figure SM3.1) and explaining 68.83% variance of the variance in the data. The stability of the three-cluster solution was validated through 5-fold cross-validation with 10 repeats, demonstrating consistent performance across folds (mean stability score = 0.51, *SD* = 0.02) and good cluster separation (mean silhouette score = 0.42, *SD* = 0.02). Silhouette scores range from -1 to 1, where scores above 0.25 indicate reasonable structure, above 0.50 suggest strong structure, and above 0.70 indicate excellent separation; in this context, our score of 0.42 indicates well-defined clusters. While a four-cluster solution offered a moderate increase in explained variance (8.6%), it showed only a negligible improvement in cluster separation (silhouette score increase of 0.01), which suggests potential overfitting. The cross-validation analysis reinforced the robustness of the three-cluster solution, showing highly consistent variance explanation across folds (*M* = 0.69, *SD* = 0.01). These metrics collectively support selecting k = 3 as the optimal solution, balancing explanatory power (68.83% variance explained) with model parsimony and cluster stability.

Figure SM3-1

Determination of optimal number of clusters using the elbow method

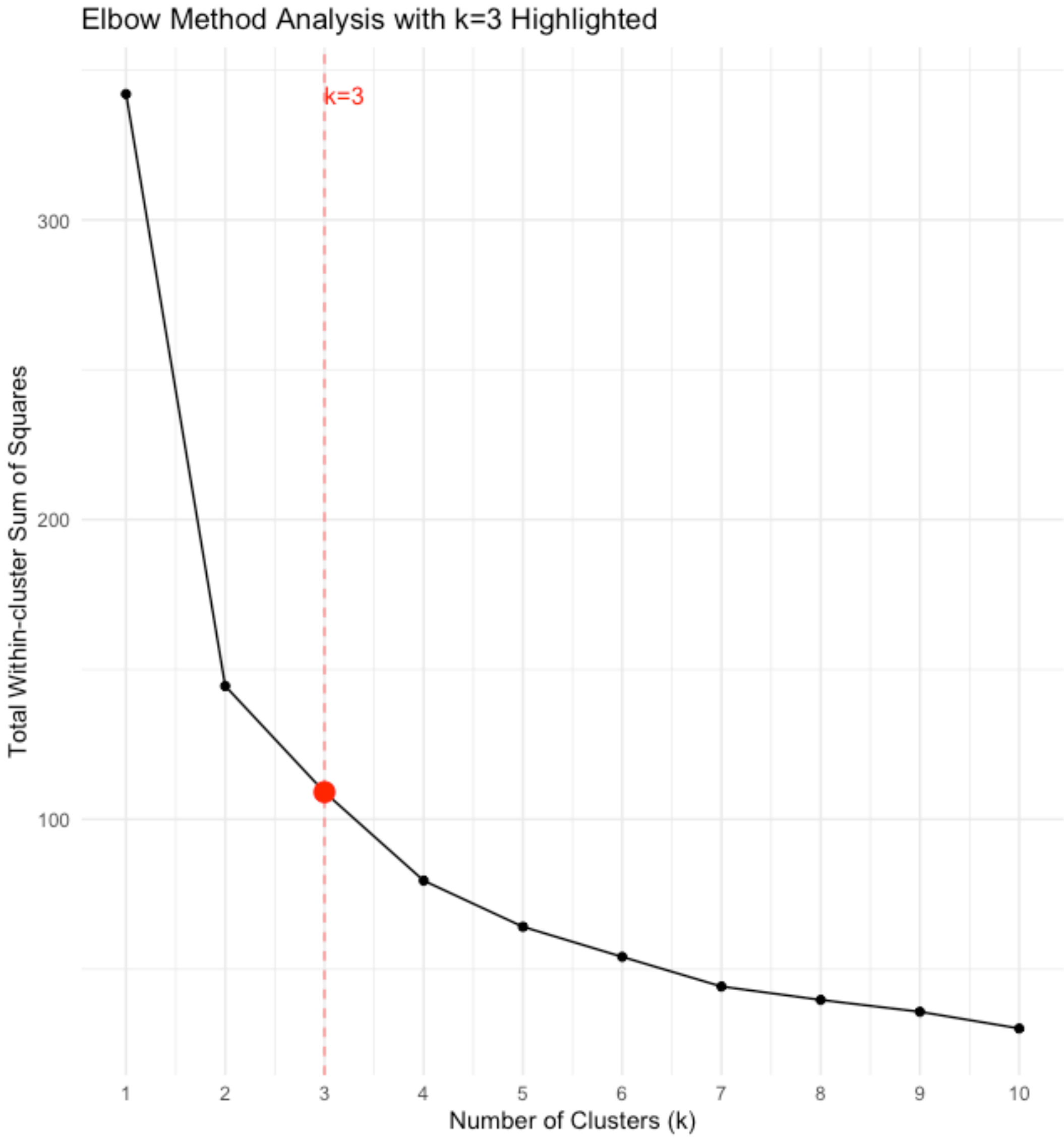

Note. The total within-cluster sum of squares (WSS) is plotted against the number of clusters (k) from 1 to 10. The optimal solution (k = 3) is highlighted in red, representing an inflection point in the WSS reduction curve. The model achieves 68.83% of the explained variance at this point, with subsequent clusters providing diminishing returns. The dashed red line indicates the selected k = 3 solution.

Supplementary Material 4 (SM4)

Continuous analysis of social autistic traits and perceptual grouping To complement the subgroup analysis reported in the main text, we examined whether perceptual grouping varied continuously with social autistic traits. For each participant we computed a continuous social-trait score as the sum of the social skill and communication subscales of the AQ (Baron-Cohen et al., 2001), the dimensions used for the subgroup clustering. We then regressed the perceptual grouping score on this continuous predictor, separately for social and geometric stimuli, controlling for stimulus order, gender, and age.

The association between social autistic traits and social perceptual grouping was not significant, b = -0.002, SE = 0.002, 95% CI [-0.005, 0.001], t(166) = -1.24, p = .215. The association with geometric perceptual grouping was likewise non-significant, b = -0.003, SE = 0.001, 95% CI [-0.006, 0.000], t(166) = -1.81, p = .073. Across the full sample, perceptual grouping therefore did not vary as a graded function of social autistic traits in either domain.

To test whether the relationship was non-linear, we refitted each model with an added quadratic term for social autistic traits (mean-centred before squaring), retaining the same covariates. The quadratic term was not significant for social grouping, b = -0.0002, SE = 0.0003, 95% CI [-0.0009, 0.0005], t(165) = -0.56, p = .576, nor for geometric grouping, b = 0.0001, SE = 0.0003, 95% CI [-0.0005, 0.0008], t(165) = 0.35, p = .726. Adding the quadratic term increased the explained variance negligibly in both domains (social: $\Delta R^2 = .002$; geometric: $\Delta R^2 < .001$). These analyses provide no evidence of either a linear or a quadratic relationship between social autistic traits and perceptual grouping across the full sample. Taken together with our subgroup analysis, these results tentatively suggest a threshold pattern rather than a graded one, with the reduced social grouping appearing to concentrate within the high-trait subgroup identified through the cluster analysis.
